# Circulating tumor DNA in appendiceal malignancies: a comprehensive review of clinical applications and limitations

**DOI:** 10.3389/fonc.2026.1935036

**Published:** 2026-09-14

**Authors:** Kellyn E. McKee, Janice Oh, Samantha M. Ruff

**Affiliations:** 1 University of Virginia School of Medicine, Charlottesville, VA, United States; 2 Department of Surgery, University of Virginia Health, Charlottesville, VA, United States

**Keywords:** appendiceal cancer, circulating tumor DNA, liquid biopsy, peritoneal metastases, peritoneal tumor DNA

## Abstract

Appendiceal cancer is a rare and biologically distinct gastrointestinal malignancy characterized by marked molecular heterogeneity and a strong propensity for peritoneal dissemination. Traditional surveillance modalities, including cross-sectional imaging and serum tumor markers, often demonstrate limited sensitivity for detecting residual or recurrent peritoneal disease, creating a need for more effective biomarkers. Circulating tumor DNA (ctDNA) has emerged as a promising noninvasive tool for genomic profiling, prognostication, and disease monitoring across gastrointestinal (GI) cancers. In this review, we synthesize current evidence regarding the application of ctDNA in appendiceal malignancies. Available studies demonstrate high concordance between plasma ctDNA and tissue-based genomic profiling, while longitudinal ctDNA monitoring shows its potential for risk stratification, recurrence detection, and postoperative surveillance, with some studies suggesting improved performance compared with conventional biomarkers. However, the clinical utility of plasma ctDNA is limited by the unique biology of appendiceal cancer, particularly its tendency toward isolated peritoneal spread and pseudomyxoma peritonei (PMP), both of which are associated with reduced systemic tumor DNA shedding and frequent false negative results. We discuss the biological mechanisms underlying these limitations and highlight peritoneal tumor DNA (ptDNA) as a promising complementary biomarker capable of overcoming the peritoneal-vascular barrier through direct sampling of the local tumor microenvironment. Emerging evidence suggests that ptDNA may improve detection of occult peritoneal disease, enhance molecular characterization, and provide prognostic information beyond that obtainable from plasma-based assays alone. While liquid biopsy technologies hold substantial promise for advancing personalized management of appendiceal cancer, prospective validation studies and standardized assay approaches are needed before widespread clinical implementation.

## 1 Introduction

Analysis of ctDNA, a subset of free DNA shed into bodily fluids by apoptotic or necrotic tumor cells, is emerging as a noninvasive biomarker for various cancers (1). In GI malignancies, a rapidly expanding body of evidence supports the utility of ctDNA for genomic profiling, therapeutic response monitoring, and the detection of minimal residual disease (2, 3). However, despite growing evidence for various applications of ctDNA, the clinical utility of ctDNA in appendiceal cancer remains understudied.

Appendiceal cancer accounts for about four percent of all GI cancers and has been increasing in incidence over the last two decades (4, 5). It is highly heterogeneous, encompassing distinct histological subtypes ranging from low-grade mucinous neoplasms to aggressive signet ring cell carcinomas, with five-year survival rates varying from 10% to 90% (6). Importantly, appendiceal tumors are molecularly distinct from colorectal cancers and exhibit a distinct tropism for peritoneal dissemination rather than hematogenous metastasis (7). This propensity for widespread abdominal seeding causes profound patient morbidity yet remains notoriously difficult to quantify. Standard cross-sectional imaging and traditional serum biomarkers such as CEA and CA125 routinely fail to detect early peritoneal recurrence (8). Plasma-based ctDNA assays have potential to fill this diagnostic void, and the goal of this review is to comprehensively synthesize current evidence evaluating the utility of ctDNA in appendiceal cancer. We also identify the biological barriers inherent to ctDNA analysis in the context of peritoneal disease and discuss future directions to optimize this biomarker for improved patient risk stratification and therapeutic management in appendiceal cancer.

## 2 Molecular landscape of appendiceal cancer

### 2.1 Subtypes of appendiceal tumors

Appendiceal neoplasms are histologically categorized into four distinct subtypes, each characterized by a unique molecular and genomic profile: epithelial neoplasms, neuroendocrine tumors, goblet cell carcinomas, and non-epithelial neoplasms. Epithelial neoplasms comprise a common class of appendiceal tumors and are further stratified into five distinct categories, including low-grade appendiceal mucinous neoplasms (LAMN), high-grade appendiceal mucinous neoplasms (HAMN), mucinous adenocarcinoma, colonic-type (non-mucinous) adenocarcinoma, and signet ring cell carcinoma (9–11). Importantly, LAMN and HAMN are biologically distinct from invasive adenocarcinomas. Although these neoplasms can disseminate mucinous tumor cells throughout the peritoneal cavity and give rise to pseudomyxoma peritonei (PMP), they generally lack infiltrative invasion and do not spread through lymphatic or hematogenous routes to cause distant metastatic disease (12). Consequently, management is centered on cytoreductive surgery with hyperthermic intraperitoneal chemotherapy (CRS-HIPEC), whereas systemic chemotherapy is typically reserved for invasive appendiceal adenocarcinomas with metastatic potential (13). In contrast, mucinous, colonic-type, and signet ring cell adenocarcinomas exhibit invasive behavior, can metastasize through lymphatic and hematogenous pathways, and are often treated using systemic therapies analogous to those employed in colorectal cancer (12). These biologic differences are particularly relevant to ctDNA analysis, as the limited vascular invasion and acellular ascites characteristic of LAMN and HAMN may reduce ctDNA shedding compared with invasive adenocarcinomas. Neuroendocrine neoplasms, historically referred to as carcinoid tumors, represent the most frequently diagnosed primary malignancy of the appendix (10). Goblet cell carcinomas are unique in that they are almost exclusively found in the appendix and clinically demonstrate characteristics of both neuroendocrine neoplasms and typical adenocarcinomas (14). Other non-epithelial neoplasms that arise from appendiceal tissue are rare and can include lymphomas and sarcomas (15). The differences between appendiceal cancer subtypes provide important context for interpreting the sensitivity and limitations of plasma-based liquid biopsy as tumor cellularity, degree of stromal and mucinous content, vascular and lymphatic invasion, and propensity for peritoneal dissemination may all influence the quantity and route of tumor-derived DNA entering the circulation.

### 2.2 Genetic drivers of appendiceal vs colorectal cancers

The molecular development of appendiceal cancer is driven by key oncogenes such as *KRAS*, *GNAS*, and *BRAF* operating in parallel with mutated tumor suppressor genes like *TP53* and *SMAD4* (16). When these alterations occur concurrently, they disrupt essential intracellular signaling pathways to accelerate cell proliferation, stimulate angiogenesis, and suppress apoptosis, ultimately driving tumor growth (17). Importantly, the mutational frequencies observed in appendiceal malignancies diverge sharply from those seen in colorectal cancers, underscoring why appendiceal tumors require a distinct clinical approach. While *KRAS* and *GNAS* mutations occur more frequently in appendiceal tumors than in colorectal cancers, several colorectal cancer driver mutations are comparatively uncommon in appendiceal disease (16). For example, genomic analyses have demonstrated markedly lower mutation rates of *TP53* (27% vs. 75%) and *APC* (8% vs. 76%) mutations in appendiceal cancers compared with colorectal cancers (17). Similarly, *PIK3CA* mutations are less frequent in appendiceal tumors (6.5%) than in colorectal cancers (17.7%) (17). In contrast, *SMAD4* mutations occur at comparable or even higher frequencies in appendiceal malignancies relative to colorectal cancer (16, 17). These molecular distinctions highlight the unique biology of appendiceal neoplasms and suggest that findings from colorectal cancer cannot always be extrapolated to appendiceal disease. The distinct genetic profiles of appendiceal cancer subtypes may also have implications for liquid biopsy, as differences in mutational burden and the prevalence of trackable tumor-specific alterations can influence the ability of plasma assays to identify and quantify ctDNA.

### 2.3 Genetic heterogeneity of appendiceal tumors

Genetic heterogeneity is also highly prevalent among individual appendiceal cancer subtypes. A molecular comparison of 53 goblet cell carcinomas against 428 appendiceal adenocarcinomas revealed that *KRAS*, *GNAS*, and *APC* are less frequently mutated in goblet cell carcinomas, whereas mutations in *CDH1*, *CHEK2*, *CDC73*, *ERCC2*, and *FGFR2* occur at a higher frequency (18). Furthermore, goblet cell carcinomas are rarely characterized by high tumor mutational burden or high-frequency microsatellite instability. Combined with the fact that only 2% of these carcinoma samples exhibit PD-L1 positivity, these findings suggest that immune checkpoint inhibitors, which are highly effective in certain colorectal cancer subsets, may lack efficacy in patients with goblet cell carcinomas (18). Non-metastatic LAMN and HAMN are also genetically distinct from more aggressive appendiceal adenocarcinoma. LAMN and HAMN are characterized by frequent *KRAS*/*GNAS* co-mutations, while mucinous adenocarcinomas acquire additional alterations such as *TP53*, *APC*, *PIK3CA*, *PTEN*, *FBXW7*, and *SMAD4* at significantly higher rates, likely contributing to their more aggressive and invasive phenotype (16).

These varying mutational patterns directly influence patient prognosis and overall survival. A study tracking 76 appendiceal cancer cases from 4 histologic subtypes including adenocarcinomas, mucinous adenocarcinomas, goblet cell carcinoid tumors, and signet ring cell carcinomas found highly favorable prognoses in patients harboring only a *GNAS* mutation, with a median overall survival of approximately 10 years (19). In contrast, patients with an isolated *TP53* mutation faced a significantly worse prognosis, with median overall survival dropping to roughly 3 years, while patients lacking either mutation fell into an intermediate category with a median overall survival of about 6 years (19). Many of these mutations, including *KRAS*, *GNAS*, and *BRAF*, can be targeted by inhibitors of key molecules in their respective pathways and thus guide management as well as prognostication (16, 20). This diversity in patient genetic profiles and their impact on survival and treatment in appendiceal cancer patients highlights a pressing clinical need for tumor-specific, biomarker-driven management, which could be aided by ctDNA analysis.

## 3 ctDNA in appendiceal cancer: current evidence

Although data regarding the clinical application of ctDNA in appendiceal malignancies remains limited, preliminary evidence highlights its significant potential for genomic profiling and risk stratification. Broadly, cell-free DNA (cfDNA) encompasses all extracellular DNA fragments circulating within the bloodstream, including ctDNA derived from tumor cells (21). While the ultimate clinical objective is often to identify and track the ctDNA fraction, many analytical platforms first extract total plasma cfDNA before sequencing it for tumor-specific mutations. Investigating this liquid biopsy approach, a comparative analysis of 56 patients evaluated matched appendiceal tumor tissue from a mixture of LAMN, mucinous adenocarcinoma, goblet cell, signet ring cell, and unspecified adenocarcinoma subtypes with plasma cfDNA from the same patients. The study revealed an overall concordance rate of 98.4% between the two modalities, with an 85.7% agreement rate specifically for therapeutically actionable alterations in *KRAS*, *GNAS*, *TP53*, and *SMAD4* (22). This study also associated specific mutations with different tumor grades. Mutations in *TP53*, *SMAD4*, and *SPTA1* were associated with intermediate- and high-grade tumors, while *GNAS* mutations were more common in low-grade disease (22). Histologic grade and cfDNA concentration independently predicted progression-free and disease-specific survival, suggesting a potential role of cfDNA profiling in tumor grade stratification and understanding disease progression (22). The feasibility of liquid biopsy has been further validated in a larger cohort of 303 appendiceal cancer patients spanning mucinous, atypical goblet cell, and adenocarcinoma subtypes, which demonstrated mutation profiles and frequencies identified via plasma ctDNA closely mirror those captured through traditional tissue-based next generation sequencing (23). Together, these findings suggest quantifying total cfDNA concentration and profiling circulating tumor-specific mutations or directly analyzing ctDNA could be a feasible ways to stratify tumor grade and track disease progression.

Liquid biopsy also shows promise as a non-invasive tool for longitudinal post-treatment surveillance. In a cohort of 94 appendiceal cancer patients including goblet cell, signet ring, and mucinous histologies, ctDNA was detectable across all tumor grades, though its detection rate was significantly diminished in patients undergoing concurrent systemic chemotherapy (8). Within this surveillance setting, ctDNA exhibited robust diagnostic performance for capturing disease recurrence following cytoreductive surgery (CRS), yielding a sensitivity of 93.8% and a specificity of 85.0% (8). It also outperformed conventional serum biomarkers including CEA, CA19-9, and CA125, serving as the sole elevated marker in 4 out of 16 cases of recurrence (8). These findings support the potential role of ctDNA as an effective surveillance tool for appendiceal cancer and provide preliminary evidence that it may enable earlier detection of recurrence than standard monitoring approaches. While these findings are promising, it is important to acknowledge that the small sample size in this study limits extrapolation to broad clinical contexts, especially given the combined cohort of heterogeneous appendiceal cancer types with unique clinical features and natural histories.

The prognostic utility of ctDNA also extends to the perioperative period. Serial ctDNA monitoring in 33 patients with colorectal or high-grade appendiceal cancer undergoing cytoreductive surgery and hyperthermic intraperitoneal chemotherapy (CRS-HIPEC) for peritoneal metastases demonstrated a significant association between rising postoperative ctDNA levels and clinical recurrence (24). Specifically, 90% of patients with increasing ctDNA trends experienced disease recurrence, compared with just 21% of those with stable or undetectable levels (24). On multivariable analysis, ctDNA emerged as the strongest independent predictor of disease-free survival, demonstrating a sensitivity of 85.0% and a specificity of 84.6% for recurrence detection (24). Notably, ctDNA elevations preceded radiographic evidence of recurrence by a median lead time of 3 months and demonstrated superior sensitivity over standard CEA testing, supporting serial ctDNA monitoring as a promising surveillance strategy following curative surgical treatment (24). These findings are supported by a prospective evaluation of preoperative plasma ctDNA in 80 patients with peritoneal carcinomatosis, the majority (73.8%) of whom had primary appendiceal malignancies. In this cohort, preoperative ctDNA levels were inversely correlated with progression-free survival, and this association remained significant after adjustment for histologic grade, with acellular peritoneal mucin and LAMN categorized as low grade and HAMN and adenocarcinomas categorized as high grade (25). These results suggest that ctDNA provides prognostic information beyond conventional histopathologic risk factors and may improve preoperative risk stratification by identifying patients who could benefit from more aggressive therapeutic approaches. Similarly, paired pre- and postoperative ctDNA measurements in 71 patients undergoing surgery for peritoneal metastases from appendiceal (64.8%), colorectal (22.5%), mesothelioma (4.2%), small bowel (4.2%), and uncategorized (4.2%) primary malignancies demonstrated that elevated ctDNA levels at either time point were associated with shorter progression-free survival, while high-grade histology, incomplete cytoreduction, and elevated preoperative ctDNA all independently predicted recurrence risk (26).

Although appendiceal-specific studies remain relatively limited, similar findings have been validated in larger colorectal cancer cohorts. Among patients with colorectal peritoneal metastases treated with CRS-HIPEC, persistent postoperative ctDNA positivity reliably identified individuals with aggressive tumor biology and an increased risk of early clinical relapse (27). Beyond the peritoneal disease setting, a prospective study of 309 patients with stage I–IV colorectal cancer demonstrated that detectable postoperative ctDNA following surgical resection was strongly associated with recurrence (28). The investigators also observed a positive correlation between tumor grade and ctDNA detection, further supporting the relationship between ctDNA positivity and aggressive disease biology (28).

Collectively, these studies suggest longitudinal perioperative ctDNA monitoring has potential for identifying patients at high risk of recurrence, refining perioperative risk stratification, and informing postoperative management (**Table 1**). However, the external validity of appendiceal-specific findings remains limited by small sample sizes and heterogeneous patient cohorts. Larger prospective, appendiceal-focused studies are therefore needed to validate these observations and define the optimal role of ctDNA in perioperative decision-making.

**Table 1:** Clinical studies evaluating ctDNA in appendiceal malignancies.

Study	Design	Cohort	Appendiceal histology	Assay	Specimen	Timing	Endpoint	Principal findings
Patel et al. (21)	Comparative study	56	LAMN, mucinous adenocarcinoma, goblet cell, signet ring cell, unspecified adenocarcinoma	Comprehensive genomic profiling (523-gene NGS panel)	Plasma cfDNA and matched tumor tissue	NR	Genomic concordance, survival	98.4% overall concordance between plasma cfDNA and tissue; 85.7% agreement for actionable alterations in KRAS, GNAS, TP53, and SMAD4. Histologic grade and cfDNA concentration independently predicted progression-free and disease-specific survival.
Shaib et al. (22)	Cohort study	303	Mucinous, atypical goblet cell, adenocarcinoma	Plasma ctDNA/NGS	Plasma and tissue	NR	Genomic profiling	Plasma ctDNA mutation profiles and frequencies closely mirrored tissue-based next-generation sequencing.
Belmont et al. (8)	Retrospective cohort	94	Goblet cell, signet ring, mucinous	Tumor-informed personalized ctDNA	Plasma ctDNA	Post-treatment, longitudinal	Recurrence detection	ctDNA was detectable across tumor grades; sensitivity 93.8% and specificity 85.0% for recurrence following CRS. ctDNA outperformed CEA, CA19-9, and CA125 and was the sole elevated marker in 4/16 recurrences.
Dhiman et al. (23)	Retrospective cohort	33	High-grade appendiceal and colorectal	Tumor-informed personalized ctDNA	Plasma ctDNA	Serial perioperative/postoperative	Recurrence, DFS	90% of patients with rising ctDNA experienced recurrence versus 21% with stable/undetectable ctDNA. Sensitivity 85.0%, specificity 84.6%; ctDNA preceded radiographic recurrence by median 3 months.
Baumgartner et al. (24)	Prospective cohort	80	Majority appendiceal (73.8%); peritoneal carcinomatosis	Plasma ctDNA	Preoperative plasma ctDNA	Preoperative	PFS, prognosis	Higher preoperative ctDNA was associated with shorter PFS after adjustment for histologic grade.
Baumgartner et al. (25)	Cohort study	71	Majority appendiceal (64.8%); also colorectal, mesothelioma, small bowel, other	Plasma ctDNA	Plasma ctDNA	Pre- and postoperative	PFS, recurrence	Elevated ctDNA at either time point was associated with shorter PFS. High-grade histology, incomplete cytoreduction, and elevated preoperative ctDNA independently predicted recurrence.

NR, not reported; LAMN, low-grade appendiceal mucinous neoplasm; NGS, next-generation sequencing; PFS, progression-free survival; DFS, disease-free survival; CRS, cytoreductive surgery.

Summary of currently available clinical studies evaluating plasma ctDNA in appendiceal malignancies, including study design, cohort characteristics, histologic composition, assay methodology, specimen and collection timing, clinical endpoints, and principal findings.

## 4 The peritoneal problem: limitations of ctDNA in appendiceal cancer metastasis

### 4.1 Biological basis for reduced ctDNA shedding in peritoneal disease

Despite the diagnostic and surveillance potential of ctDNA in appendiceal oncology, a critical biological hurdle restricts its clinical sensitivity: the poor systemic shedding characteristic of peritoneal disease. Because tumor-derived DNA must cross the peritoneal mesothelium, submesothelial stroma, and capillary endothelium before reaching the bloodstream, peritoneal tumors have substantially less access to the systemic circulation than highly vascularized visceral metastases (**Figure 1**) (29). The unique biology of PMP is also characterized by low tumor cellularity, abundant extracellular mucin, and minimal vascular invasion, which could all contribute to confinement of disease to the peritoneal cavity and limit release of tumor DNA into the systemic circulation (30). Consequently, malignancies demonstrating localized peritoneal tropism, such as peritoneal colorectal or appendiceal carcinomas, display a consistently depressed systemic ctDNA footprint compared with visceral or nodal metastases or recurrences (29). A retrospective case series of 13 patients with isolated peritoneal disease, including 8 with appendiceal cancer, highlights the challenges of ctDNA detection in peritoneal-only malignancies. While ctDNA was detectable in all patients with gastric cancer, only 37.5% of appendiceal cancer patients had detectable ctDNA at baseline (31). Despite these low concentrations, serial ctDNA measurements tracked with changes in disease burden during treatment and, in some cases, facilitated earlier detection of recurrence (31). These findings highlight the importance of highly sensitive, tumor-informed assays in appendiceal cancer, as broader panel-based ctDNA approaches may lack sufficient sensitivity to detect disease in many patients with isolated peritoneal involvement.

**Figure 1:**
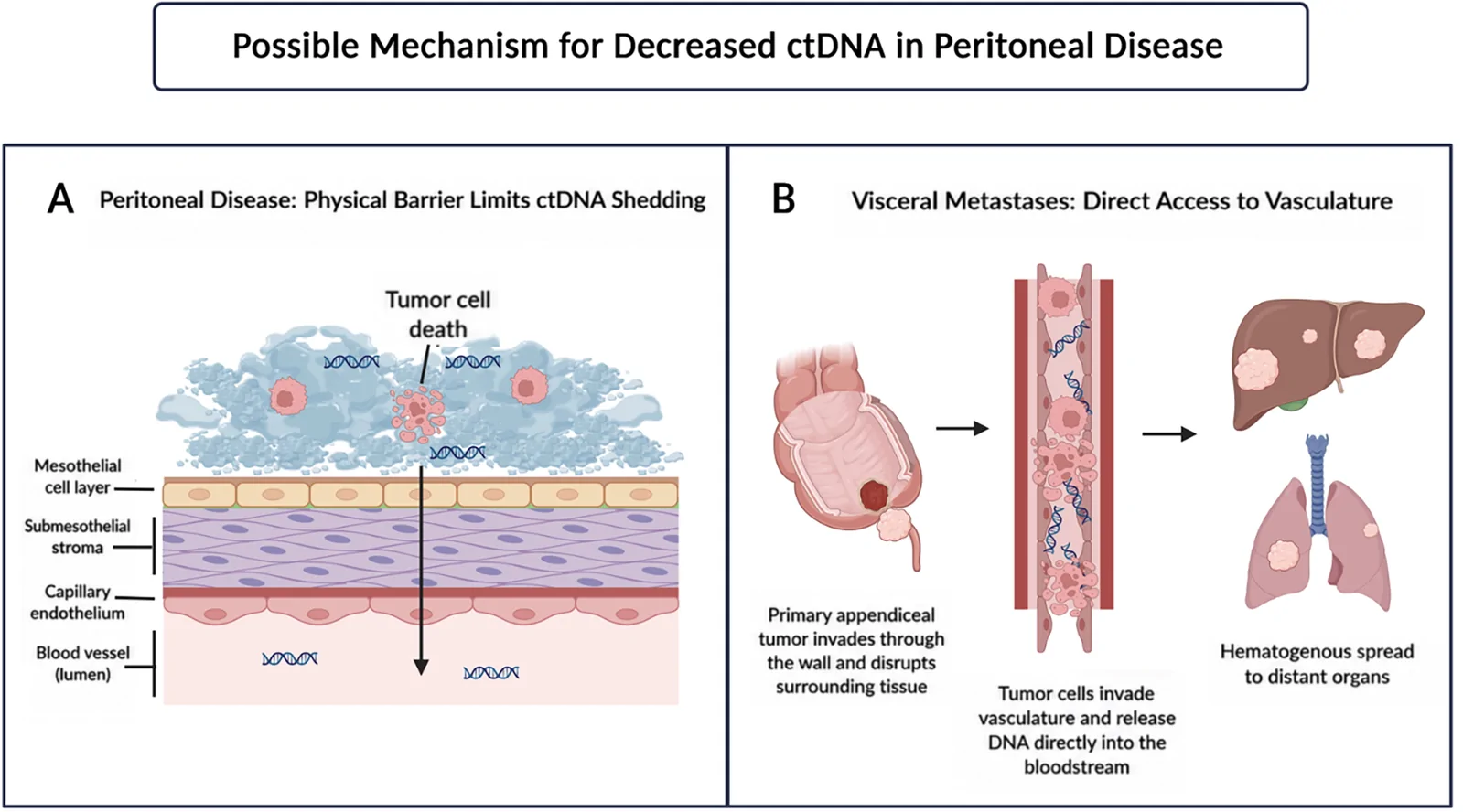
Peritoneal metastases **(A)**, including PMP, may exhibit lower systemic ctDNA levels than visceral metastases **(B)** due to both anatomical and biological factors. Tumor-derived DNA released within the peritoneal cavity must traverse multiple physical barriers, including the peritoneal mesothelium, submesothelial stroma, and capillary endothelium, before entering the systemic circulation, limiting ctDNA dissemination into peripheral blood. In addition, PMP is characterized by low tumor cellularity, abundant extracellular mucin, and minimal vascular invasion compared to visceral metastases, all of which may further restrict tumor DNA release and vascular access. Together, these features promote confinement of DNA to the peritoneal cavity and contribute to the consistently reduced systemic ctDNA footprint observed in peritoneal colorectal and appendiceal malignancies compared with highly vascularized visceral or nodal metastases. Created in BioRender. Ruff, S. (2026) https://BioRender.com/gdgv1p0.

This physiological constraint is uniquely relevant to appendiceal cancer management, given the propensity of certain appendiceal neoplasms for peritoneal dissemination. In particular, mucinous appendiceal neoplasms, including LAMN and HAMN, characteristically disseminate within the peritoneal cavity following transmural extension or rupture (9). The peritoneum also serves as the primary site for treatment failure and post-therapeutic disease recurrence (32). As such, finding strategies to augment the utility of ctDNA in peritoneal disease is of particular importance in appendiceal cancer.

### 4.2 ctDNA detection in peritoneal carcinomatosis and pseudomyxoma peritonei

This diminished peritoneal shedding pattern is corroborated across wider gastrointestinal cancer cohorts. A retrospective evaluation of 279 patients with advanced colorectal, esophageal, gastric, small bowel, or pancreaticobiliary cancer confirmed that ctDNA detection is more challenging in the setting of isolated peritoneal carcinomatosis than in visceral metastatic disease (33). Across diverse primary tumor types, stage IV patients with isolated peritoneal metastases exhibited significantly reduced plasma ctDNA concentrations compared to those with visceral metastases, resulting in a lower rate of detection of actionable somatic variants via matched tissue-plasma sequencing (33).

This biological barrier is even more pronounced in PMP, a condition characterized by massive mucinous ascites most often caused by appendiceal mucinous neoplasms (30). One multicenter retrospective study of 20 patients with PMP of predominantly appendiceal origin evaluated KRAS and GNAS mutations in mucinous tumor samples alongside plasma cfDNA using different analytical platforms across three participating research units (34). In two units, KRAS and GNAS mutations were assessed using droplet digital polymerase chain reaction, while the third unit used next-generation sequencing platform for plasma cfDNA analysis. Despite confirmed KRAS mutations in 15 of 20 mucinous tumor samples, no corresponding KRAS mutations were detected in peripheral blood across the cohort (34). While interesting preliminary findings, it is important to note that this study is significantly limited by its small sample size and unspecified appendiceal cancer subtypes. A larger multi-institutional study published in 2026 found a similar trend, detecting ctDNA in only 8% of 95 PMP patients of primarily (88%) appendiceal origin, including LAMN, HAMN, mucinous adenocarcinoma, and goblet cell carcinoid tumors (35). Plasma ctDNA testing also detected less than 10% of known *KRAS* or *GNAS* mutations in these patients, reinforcing the challenges of using ctDNA for disease monitoring or molecular profiling in PMP (35). The researchers did not break down the data by specific appendiceal cancer subtype and, given the heterogeneous nature of appendiceal tumors, the conclusions that can be drawn from this data and applied to patients with any given appendiceal cancer subtype are somewhat limited. However, these findings highlight an important limitation of liquid biopsy in peritoneal malignancies and suggest that negative ctDNA results should be interpreted cautiously, as reduced sensitivity may lead to false-negative findings and underdetection of clinically relevant mutations.

## 5 Peritoneal tumor DNA as an alternative to ctDNA

To circumvent the systemic sensitivity limitations imposed by the peritoneal-vascular barrier, attention has turned toward analyzing peritoneal tumor DNA (ptDNA). Obtained directly from regional fluid compartments via paracentesis or peritoneal lavage, ptDNA reflects the immediate shed of tumor-derived nucleic acids into the local microenvironment, yielding substantially higher target concentrations than matched peripheral blood samples (36). Early studies suggest that ptDNA may overcome many of the sensitivity limitations associated with the plasma-peritoneal barrier. Limited data exists for appendiceal cancer alone, but a comprehensive systematic review evaluating the utility of ptDNA collected from ascites or peritoneal washings across diverse gastrointestinal and gynecological malignancies confirmed that regional liquid biopsy signaling was associated with advanced disease stages and poor clinical outcomes (37). In gastric cancer, the most robustly characterized cohort, ptDNA positivity strongly correlates with higher stage disease, increased risk of peritoneal metastases, and shorter progression-free and overall survival (37). Similar adverse prognostic trends are emerging in colorectal, pancreatic, and endometrial malignancies (37). Overall, this review identified ptDNA as a promising prognostic biomarker with potential advantages over plasma ctDNA for peritoneal malignancies while also highlighting substantial heterogeneity in sample collection and processing across studies, underscoring the absence of standardized approaches for obtaining and analyzing peritoneal fluid specimens. Ascites or mucinous peritoneal fluid is typically collected by paracentesis or aspiration in patients with established peritoneal disease, whereas peritoneal lavage specimens are typically obtained intraoperatively by instilling fluid into the peritoneal cavity and subsequently collecting the washings. Processing protocols also vary across studies, including differences in centrifugation procedures, sample volumes, storage conditions, and DNA extraction methods.

It is also important to distinguish the differing potential clinical applications of peritoneal washings and ascites, which are both included in this review (37). Peritoneal washings are typically collected intraoperatively and may provide information about occult peritoneal disease that can complement surgical assessment and potentially inform cytoreductive decision-making (38). In contrast, ascites sampling is generally performed in patients with established or advanced peritoneal disease and may provide a readily accessible source of tumor DNA for molecular characterization, including assessment of tumor heterogeneity, clonal evolution, and potential resistance mechanisms (38). This distinction means that while ascites-derived ptDNA may have limited utility for initially diagnosing the presence of peritoneal disease, it may have distinct value for longitudinal molecular monitoring of advanced disease.

Improved diagnostic performance of peritoneal cfDNA assays compared to standard diagnostic modalities has also been demonstrated in prospective clinical cohorts. In an evaluation of patients with colorectal malignancies, peritoneal cfDNA collected from peritoneal lavage achieved 100% sensitivity and 77.3% specificity for identifying biopsy-confirmed peritoneal metastases, a diagnostic accuracy further sustained in an independent validation cohort (39). Notably, approximately 22% of patients without clinically detectable peritoneal metastases had elevated peritoneal cfDNA levels, raising the possibility that total peritoneal cfDNA can identify occult peritoneal micro-metastases that are missed by conventional staging methods (39). Longitudinal tracking further revealed that positive peritoneal cfDNA signatures were significantly associated with decreased recurrence-free survival and frequently predate radiographic evidence of recurrence (39).

In addition to its promise for diagnosis and recurrence monitoring, ptDNA may also aid molecular profiling and targeted therapy selection. A pilot study evaluating ptDNA in malignant ascites and peritoneal lavage samples from 37 patients with peritoneal carcinomatosis from appendiceal (including mucinous adenocarcinoma, signet ring, and goblet cell subtypes), colorectal, pancreatic, and small bowel primary malignancies identified *KRAS*-mutant DNA in all patients with known *KRAS* mutations (40). *KRAS* mutations were also detected in a subset of patients whose tissue testing had been *KRAS* wild-type, suggesting that ptDNA may uncover tumor heterogeneity or subclonal mutations missed by conventional tissue sampling (40). Importantly, lower mutant allele frequencies were associated with disease amenable to cytoreductive surgery, whereas higher levels correlated with unresectable disease, indicating that ptDNA may serve as a biomarker to help guide preoperative surgical decision-making in patients with peritoneal disease (40). However, this study was exploratory, and findings did not change patient management, so further studies investigating the clinical application of ptDNA to treatment stratification are needed to elucidate the clinical utility of these findings.

Despite some promising initial evidence, there are several important limitations to consider when evaluating the clinical utility of ptDNA assays. Patients presenting with ascites most likely already have clinically significant disease, and without pockets of ascites available for ptDNA collection, standard practice is to use peritoneal washings. Peritoneal lavage is performed during abdominal surgery by instilling fluid into the peritoneal cavity and subsequently collecting the fluid, which is far more invasive than a peripheral blood test and has the potential to dilute free DNA levels during collection (36). Furthermore, there are currently no widely adopted guidelines standardizing the collection, processing, or analytical methods for peritoneal lavage or ascites specimens used for ptDNA analysis. Existing studies differ in sample collection techniques, fluid volumes, processing protocols, storage conditions, and molecular assays, limiting direct comparison of results across cohorts. Certain appendiceal tumor types, such as LAMN and HAMN, often generate acellular mucin in the peritoneum, which may increasingly limit the utility of ptDNA in these patients (22). This further highlights the need for trials evaluating the potential role of ctDNA and ptDNA in different appendiceal cancer subtypes and for standardized protocols to establish the optimal methods for ptDNA collection and analysis.

## 6 Conclusion

Liquid biopsy using ctDNA represents a highly promising, minimally invasive frontier in the clinical management of appendiceal cancer. Current evidence establishes that plasma-based ctDNA assays possess high genomic concordance with matched tumor tissue, offering an alternative strategy for molecular profiling and grade stratification. In post-treatment and perioperative settings, longitudinal ctDNA monitoring may enable earlier detection of recurrence than conventional serum biomarkers and cross-sectional imaging in some patients. However, these findings remain preliminary and have not been validated in prospective comparative studies demonstrating improved clinical outcomes with ctDNA-guided monitoring. The unique biology of appendiceal malignancies also presents a formidable challenge to systemic liquid biopsies. The physical constraint of the peritoneal-vascular barrier, alongside the low cellularity and thick extracellular mucin characteristic of PMP minimizes systemic tumor shedding. Consequently, relying solely on peripheral blood ctDNA carries a risk of false-negative results in patients with isolated peritoneal disease. Regional ptDNA assays are an emerging alternative with the potential to overcome this barrier. By sampling directly from the local microenvironment, ptDNA bypasses the anatomical barriers of the peritoneum, demonstrating superior diagnostic sensitivity for detecting occult micro-metastases. Ultimately, while plasma ctDNA is a promising tool for tracking aggressive or systemic disease, integrating local fluid-based biomarkers like ptDNA appears essential to optimizing the sensitivity of liquid biopsy for the entire spectrum of appendiceal malignancies.

## 7 Future directions

Despite encouraging preliminary results, evidence supporting ctDNA and ptDNA in appendiceal cancer remains limited, consisting primarily of retrospective case series, small institutional cohorts, and mixed gastrointestinal cancer populations. Because appendiceal cancer is rare, studies frequently combine distinct histologic subtypes despite their marked biological and clinical differences, limiting subtype-specific conclusions. Large, prospective, multi-institutional studies are needed to validate these biomarkers, define their clinical utility across individual histologies, and support their integration into clinical guidelines. To date, there are also no prospective clinical trials evaluating ctDNA-guided treatment escalation or de-escalation in appendiceal oncology or comparing outcomes between patients monitored with conventional imaging and serum biomarkers versus those monitored with ctDNA. Future trials designed to test whether the persistence of postoperative ctDNA or ptDNA signals can safely guide the intensity or duration of adjuvant systemic chemotherapy following CRS and HIPEC are indicated to solidify the utility of these tools in clinical practice. Maximizing the diagnostic accuracy of liquid biopsies will also likely require moving beyond isolated DNA tracking. Future strategies should explore a multifaceted approach, combining ctDNA and ptDNA analysis with other circulating analytes, such as cell-free tumor RNA, exosome-based biomarkers, and localized peritoneal metabolites to create even more sensitive diagnostic assays. The utility of tumor-derived DNA in appendiceal cancer is currently critically understudied, particularly given the preliminary body of literature suggesting further investigation may inform future diagnostic, surveillance, and clinical management strategies for patients with appendiceal malignancies.
